# Identification of the Early Stage of Alzheimer's Disease Using Structural MRI and Resting-State fMRI

**DOI:** 10.3389/fneur.2019.00904

**Published:** 2019-08-30

**Authors:** Seyed Hani Hojjati, Ata Ebrahimzadeh, Abbas Babajani-Feremi

**Affiliations:** ^1^Department of Pediatrics, University of Tennessee Health Science Center, Memphis, TN, United States; ^2^Department of Electrical Engineering, Babol University of Technology, Babol, Iran; ^3^Neuroscience Institute and Children's Foundation Research Institute, Le Bonheur Children's Hospital, Memphis, TN, United States; ^4^Department of Anatomy and Neurobiology, University of Tennessee Health Science Center, Memphis, TN, United States

**Keywords:** Alzheimer's disease (AD), mild cognitive impairment (MCI), resting-state fMRI, graph theory, machine learning, hub nodes

## Abstract

Accurate prediction of the early stage of Alzheimer's disease (AD) is important but very challenging. The goal of this study was to utilize predictors for diagnosis conversion to AD based on integrating resting-state functional MRI (rs-fMRI) connectivity analysis and structural MRI (sMRI). We included 177 subjects in this study and aimed at identifying patients with mild cognitive impairment (MCI) who progress to AD, MCI converter (MCI-C), patients with MCI who do not progress to AD, MCI non-converter (MCI-NC), patients with AD, and healthy controls (HC). The graph theory was used to characterize different aspects of the rs-fMRI brain network by calculating measures of integration and segregation. The cortical and subcortical measurements, e.g., cortical thickness, were extracted from sMRI data. The rs-fMRI graph measures were combined with the sMRI measures to construct input features of a support vector machine (SVM) and classify different groups of subjects. Two feature selection algorithms [i.e., the discriminant correlation analysis (DCA) and sequential feature collection (SFC)] were used for feature reduction and selecting a subset of optimal features. Maximum accuracy of 67 and 56% for three-group (“AD, MCI-C, and MCI-NC” or “MCI-C, MCI-NC, and HC”) and four-group (“AD, MCI-C, MCI-NC, and HC”) classification, respectively, were obtained with the SFC feature selection algorithm. We also identified hub nodes in the rs-fMRI brain network which were associated with the early stage of AD. Our results demonstrated the potential of the proposed method based on integration of the functional and structural MRI for identification of the early stage of AD.

## Introduction

Alzheimer's disease (AD) is a neurodegenerative disorder, known as a disconnection syndrome that disturbs communication between different brain regions (1). It implies that the brain network is changed during the transition from healthy condition to mild cognitive impairment (MCI) and AD. Since intervention prior to the occurrence of overt and irreversible neuronal loss is critical for the maintenance of normal brain function, prediction of MCI, and conversion to AD in cognitively normal older adults is a priority for AD research.

Approximately 15% of adults older than 65 years old suffer from MCI and from these more than half progress to AD within 5 years (2). Prediction of early stage of AD is important and several studies have focused on investigating this prediction. Structural magnetic resonance imaging (sMRI) can be utilized to reliably characterize brain volumes, areas, cortical thickness, and curvature (3), and has been widely used to investigate alteration of these brain measures in transition from normal aging to AD. Patients with AD have diminished memory and executive function. Patients with early stage AD can have MCI, and although impaired, may perform similarly to normal older adults on easier memory tasks. Prognostic predictions of subjects from prodromal stages such as MCI is an area of great clinical interest (4, 5). However, prediction of symptomatic progression remains a relatively unexplored task. Memory impairment and dementia are common in the elderly population. Prognostic forecasting of symptom severity is complicated not only by the heterogeneity in demographics and clinical presentation, but also highly variable and non-linear symptom patterns exhibited in those suffering from MCI (6, 7). In a recent study, Eskildsen et al. used patterns of cortical thickness and identified cortical regions potentially discriminative for separating MCI patients into converters (MCI-C), who received a diagnosis of AD dementia within 2 years, and non-converters (MCI-NC), who remained stable for 3 years (8). They reported promising results for the prediction of patients with prodromal AD progressing to probable AD. Eskildsen et al. considered “time to conversion” and separated patients in different groups based on this time, and reported <80% accuracy for predicting conversion to AD. Beheshti et al. utilized a voxel based morphometric technique to extract the global and local gray matter volumes, and then used these volumes to classify AD and healthy controls (HC) (9).

Several studies have investigated AD induced alterations of the brain network using the resting-state functional MRI (rs-fMRI) (10–16). rs-fMRI has been shown to be a powerful tool for identifying the pathophysiology of functional connectivity not only in patients with AD but also in patients with other neurological or neuropsychiatric conditions (17). Accumulating evidence suggests that intrinsic connectivity at rest provides the communication channels of task information (18). rs-fMRI networks have been shown to be highly sensitive to AD (19). We and other investigators have reported the ability of rs-fMRI in identification of patients with MCI and AD (13–15, 20). We demonstrated potential of rs-fMRI in prediction of the early stage of AD (10). Grieder et al. suggested that cognitive decrease symptoms in AD is directly related to reduction of complexity in the brain network (21). It was reported that rs-fMRI functional connectivity can show AD-related cognitive impairment in an aging population with health, MCI, and AD (16).

Neuroimaging modalities, such as positron emission tomography (PET), diffusion-tensor imaging (DTI), rs-fMRI, and sMRI, have been found informative in providing biomarkers of conversion from MCI to AD (22–27). While most of previous studies considered a single modality approach for diagnosis of AD (28), it is expected that a multi-modal approach can improve accuracy of prediction of conversion to AD compared to a single-modality approach (29). Tong et al. utilized features extracted from sMRI, PET, cerebrospinal fluid (CSF) biomarkers, and categorical genetic information, and classify HC, MCI and AD with an accuracy of 60.2% (29). Peng et al. developed a kernel-learning-based method to combine sMRI, PET, and genetic information for AD and MCI diagnosis, and reported classification accuracies of 96.1%, 80.3%, and 76.9% for AD vs. HC, MCI vs. HC, and AD vs. MCI, respectively (30). Ahmed et al. combined DTI with sMRI to improve accuracy of AD, MCI and HC classification, and obtained an accuracy between 76% (AD vs. MCI) to 90% (AD vs. HC) for two-group classification. Dyrba et al. applied a multimodal approach based on sMRI, DTI, and rs-fMRI and classify AD from HC with an area under curve (AUC) of the receiver operating characteristic of 82% (31).

The brain topology analysis based on the graph theory provides powerful tools to study structural and functional characteristics of the brain network. Graph theory is a mathematical tool that is capable of concisely quantifying the properties of complex systems and modeling interrelationships between the brain regions. Since a large number of local and global graph measures, i.e., features, can be extracted from the brain networks, reducing dimension of features is an essential process for identifying optimal subset of features. In this study, we employed two feature selection algorithms in a machine learning approach to identify discriminative features for classifying AD, MCI, and normal aging. We developed an automatic classification algorithm that combined information from sMRI with rs-fMRI graph measures to classify four groups of subjects (AD, MCI-C, MCI-NC, and HC). We used baseline rs-fMRI and sMRI data for MCI-C and MCI-NC patients. The MCI-NC patients did not convert to AD in 36 months after the baseline rs-fMRI, although MCI-C patients converted to AD from 6 to 36 months after the baseline rs-fMRI. We did not use the “time to conversion” of MCI-C patients in our algorithm to test performance of the proposed method in a challenging condition where this information is unknown in real clinical application. Our aim was to develop a method with an ability to distinguish potential “MCI-decliners” from those who remain stable. To our knowledge, this is the first study that investigated integration of rs-fMRI and sMRI for four-group classification (i.e., AD, MCI-C, MCI-NC and HC).

## Materials and Methods

### Overall Procedure

**Figure 1:**
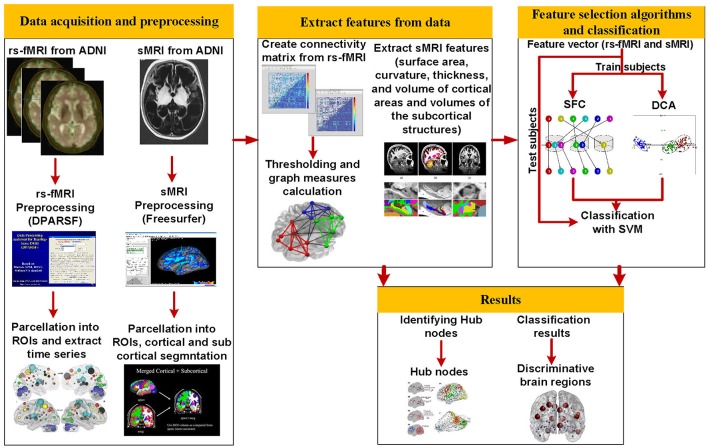
The overall procedures of this study.

The overall procedure of our proposed method is shown in Figure 1. Structural MRI (T1-weighted images) and rs-fMRI data of 177 subjects (34 AD, 25 MCI-C, 69 MCI-NC, and 49 HC) were used in this study. After preprocessing of rs-fMRI data, we used the Dosenbach atlas (32) to parcellate the brain into 160 region of interests (ROIs), and the adjacency matrix was calculated using the Pearson correlation between the time series of each pair of brain regions. We calculated 10 local and 13 global graph measures based on the adjacency matrix in each patient. FreeSurfer was utilized for preprocessing, cortical reconstruction, and volumetric segmentation of sMRI images. Volumes of the subcortical structures in addition to the surface area, curvature, thickness, and volume of 148 cortical areas, based on the Destrieux atlas (33), were calculated and used as sMRI features in our algorithm. The rs-fMRI local and global graph measures were combined with sMRI measures to generate a feature vector in each patient. Two feature selection algorithms were applied to find an optimal subset of features for support vector machine (SVM) (10). We trained, cross-validated, and tested SVM to classify AD, MCI-C, MCI-NC, and HC using the selected rs-fMRI and sMRI features. We performed a second analysis on rs-fMRI data to identify hub nodes of the brain network and identify alteration of hubs in transient from healthy aging to AD.

### Subjects

We included 34 patients with AD (average age 72.5 years, 18 female), 25 patients with MCI-C (average age 73 years, 11 female), 69 patients with MCI-NC (average age 72.9 years, 37 female), and 49 HC (average age 74.4 years, 28 female) from Alzheimer's Disease Neuroimaging Initiative (ADNI) database in this study. Subjects of this study were selected based on availability of both rs-fMRI and sMRI datasets in ADNI. We used the diagnosis variables to select the converted and non-converted subjects. In the current study, we tried to include all subjects, listed in ADNI database and had a complete set of sMRI and rs-fMRI data. The MCI-C patients were converted to AD between 6 and 36 months. The MCI-NC patients did not convert to AD after 36 months of follow-up. The patients with AD had a Mini-Mental State Examination (MMSE) score of 20–26, a Clinical Dementia Rating (CDR) of 0.5 or 1.0, and met the National Institute of Neurological and Communicative Disorders and Stroke and the AD and Related Disorders Association (NINCDS/ADRDA) criteria for probable AD. The patients with MCI had MMSE scores between 24 and 30, a memory complaint, objective memory loss measured by education adjusted scores on Wechsler Memory Scale Logical Memory II, a CDR of 0.5, absence of significant levels of impairment in other cognitive domains, essentially preserved activities of daily living, and an absence of dementia. The normal subjects were non-depressed, non-MCI, and non-demented, and had a MMSE score of 24–30 and a CDR close to zero. Demographic information of subjects is summarized in Table 1. Subjects for this study were selected based on availability of both rs-fMRI and sMRI datasets.

**Table 1:** Demographic and clinical information.

	**HC**	**MCI-NC**	**MCI-C**	**AD**	***P*-value**
Number	49	69	25	34	
Male/Female	21/28	32/37	14/11	16/18	0.76^a^
Age	74.47 ± 7.68	72.95 ± 11.92	73.02 ± 11.80	72.54 ± 7.02	0.81^b^
MMSE score	29.35 ± 1.63	27.57 ± 2.21	26.64 ±1.85	21.24 ± 3.37	0.0003^b^
CDR score	0.035 ± 0.21	0.5 ± 0.0	0.5 ± 0.0	0.92 ± 0.31	0.0001^b^

MMSE, mini-mental state examination; CDR, clinical dementia rating;

a Fisher extract test;

b *ANOVA test*.

### Data Acquisition and Preprocessing

The functional and structural MRI images were collected according to the ADNI acquisition protocol (34)^1^. A total of 140 functional volumes (TR/TE 3000/30 ms, flip angle = 80°, 3.313 mm slice thickness, 48 slices) were obtained. Standard preprocessing routines were applied on rs-fMRI dataset using Data Processing Assistant for Resting State fMRI (DPARSF) package (35) and SPM12 toolbox (http://www.fil.ion.ucl.ac.uk/spm). Slice-timing correction to the last slice was performed. The fMRI time-series realigned using a six-parameter rigid-body spatial transformation to compensate for head movement effects. Then all images were normalized into the Montreal Neurological Institute (MNI) space, resampled to 3-mm isotropic voxels, detrended, smoothed using a Gaussian filter with FWHM = 4 mm, and band-pass filtered (0.01–0.08 Hz). To reduce the effect of the physiological artifacts, the whole-brain signal was removed by a multiple linear regression analysis. In addition to the global mean signal, six head motion parameters, the cerebrospinal fluid (CSF), and the white matter signals were removed as nuisance covariates to reduce the effects of motion and non-neuronal BOLD fluctuations (36).

T1-weighted MRI of all subjects were processed using FreeSurfer (version 4.5.0). Cortical reconstruction and volumetric segmentation were performed using the following procedures: removal of non-brain tissue using a hybrid watershed/surface deformation procedure (37); automated Talairach transformation; segmentation of the subcortical white matter and deep gray matter volumetric structures (including hippocampus, amygdala, caudate, putamen, and ventricles) (37–39); intensity normalization; tessellation of gray matter and white matter boundary; automated topology correction (40); and deformation following intensity gradients to optimally place the gray/white and gray/cerebrospinal fluid borders at the location where the greatest shift in intensity defines the transition to the other tissue class (41, 42). After completing the cortical models, registration to a spherical atlas was performed (43), followed by cortical parcellation based on Destrieux atlas (33), and subcortical segmentation.

### Extracting Features From rs-fMRI and sMRI Data

An adjacency matrix was calculated using the Pearson's correlation between the time series of the fMRI signals of all pairs of 160 ROIs of Dosenbach atlas. We used a similar method detailed in our previous studies (12–15) and converted the weighted adjacency matrices to binary ones by applying an optimal threshold (44). By maximizing the global cost efficiency (GCE) of the brain network (44), we identified an optimal threshold for each adjacency matrix. To have the same number of connections after thresholding of the adjacency matrices of all subjects, we used an average optimal threshold across subjects at 19.2%, and then computed the graph measures. Then 10 local and 13 global graph measures were calculated based on rs-fMRI adjacency matrix. The local graph measures were betweenness centrality, clustering coefficient, characteristic path, community structure Newman, community structure Louvain, eccentricity, eigenvector centrality, rich club coefficient, sub graph centrality, and participation coefficient (45). The global graph measures were assortativity, clustering coefficient, characteristic path, community structure Newman output, community structure Louvain output, cost efficiency (two measures), density, efficiency, graph radius, graph diameter, transitivity, and small-worldness (45).

The surface area, average cortical curvature, average and standard deviation of thickness, and volume of gray matter of 148 cortical areas (according to Destrieux atlas) were considered as sMRI features in our algorithm. We also considered 34 features corresponding to volumes of subcortical structures in our algorithm. To make measurements comparable between subjects, an anatomical normalization was performed. For each subject, all volume quantifications were divided by the corresponding estimated intracranial volume (eTIV) and area quantifications were divided by the total area of the same hemisphere. Neither cortical thickness nor curvature needed to be anatomically normalized. After the feature extracting step, the features were normalized in each subject individually.

### Feature Selection

Solving pattern recognition or classification problems with data of high dimensionality is a challenging issue, particularly in neuroimaging applications with limited samples, and large number of features. The learning models tend to overfit and become less generalizable if input features are redundant or irrelevant to classification. Feature selection is usually performed to identify relevant features, reduce dimensionality of the trained model, and improve generalization of the model (12). An efficient feature selection algorithm is the essential part of a machine learning approach in case of high dimensional features. We utilized two feature selection algorithms in this study: discriminant correlation analysis (DCA) and sequential feature collection (SFC). We have shown efficiency of the SFC feature selection algorithm in identifying the early stage of AD (10, 11). Efficiency and reliability of the DCA feature selection algorithm have also been demonstrated in several previous studies (46, 47).

DCA has been used in pattern recognition applications for fusing the features extracted from multiple modalities or combining different feature vectors extracted from a single modality (48). DCA has a low computational load and can be employed in real-time applications. DAC is a variant of principal component analysis (PCA). PCA is the most prominent tool for reducing size of a high-dimensional feature vector, especially in unsupervised learning. DCA was developed for supervised learning environment, as a supervised PCA, to maximize the discriminant capability of classification (49). The DCA transforms features space into signal and noise subspaces. The signal subspace of DCA is associated with classification effectiveness and the noise subspace is not related to the discriminant power of the classification. For a feature vector in DCA, a within-class and a between-class scatter matrices are constructed to represent the noise and signal subspaces, respectively. Then transformed features to signal space are calculated by maximizing a signal-to-noise ratio based on the within-class and between-class scatter matrices (49). The DCA can also perform an effective feature fusion by maximizing the pairwise correlations across the two feature sets and, at the same time, eliminating the between-class correlations and restricting the correlations to be within the classes.

We developed the SFC algorithm to find an optimal subset of features (with a small number of features) (10). The SFC algorithm sorts all features using the multivariate minimal redundancy maximal relevance (MRMR) feature selection algorithm. The MRMR feature selection algorithm selects features that have maximal statistical dependency based on mutual information by considering relevant and redundant features simultaneously (50). The MRMR is defined as:

(1) $$\begin{array}{r} MRMR=MAX_{s}\left\{\frac{1}{\left|S\right|}\underset{f_{i}\epsilon S}{\sum }I\left(f_{i};c\right)-\;\frac{1}{|S{|}^{2}}\underset{f_{i},f_{j}\epsilon S}{\sum }I\left(f_{i};f_{j}\right)\right\} \end{array}$$

where the relevance of a feature set *S* for class *C* is defined by the average value of mutual information *I*(.,.) between the individual feature *f*_*i*_ and the class *C*, and the redundancy of all features in the feature set *S* is the average value of mutual information between the features *f*_*i*_ and *f*_*j*_. The SFC algorithm is described in detail previously (10). Briefly, features were first sorted based on their MRMR scores. The first and the last features in the sorted feature vector had maximum and minimum discrimination ability, respectively, in classification. Then a combination of filter and wrapper feature selection algorithms were used to find optimal subset of features with best classification accuracy.

### Classification

To evaluate performance of the prosed method for classification of four groups (AD, MCI-C, MCI-NC, and HC), we used the *k*-fold cross-validation (KCV) which is one of the most widely used resampling techniques (51), and its estimates for the cross-validation errors nearly agree with the true errors (52). In addition, we evaluated performance of our classification algorithm using independent and non-training test samples. To this end, we used a 5-fold approach and assigned 80% of subjects in each of four groups to train/cross-validation set (*n* = 141; 27 AD, 20 MCI-C, 55 MCI-NC, and 39 HC) and 20% of subjects to independent and non-training test set (*n* = 36; 7 AD, 5 MCI-C, 14 MCI-NC, and 10 HC). We then used another 5-fold for cross-validation and further divided the train/cross-validation set to 80% for training (*n* = 113; 22 AD, 16 MCI-C, 44 MCI-NC, and 31 HC) and 20% for cross-validation (*n* = 26; 5 AD, 4 MCI-C, 11 MCI-NC, and 8 HC). We used SFC or DCA algorithms for feature selection based on a combined sMRI and rs-fMRI features of *n* = 141 subjects in train/cross-validation set. An unequal sample size may cause bias in results of a classifier. We prevented this possible bias by using equal number of training and test samples in four groups based on a similar approach described in details in our previous study (11). Since MCI-C group has minimum number of subjects, we randomly selected a subset of subjects in HC, MCI-NC, and AD groups equal to the number of MCI-C subjects. This random selection was repeated 1,000 times and average performance of the classifier across this repetition was calculated.

The selected features were used to train and cross-validate an SVM to classify four groups of subjects (AD, MCI-C, MCI-NC, and HC) in the train/cross-validation set. We used SVM for classification in this study. The SVM classifier was implemented in MATLAB using LIBSVM toolbox (53). After training and cross-validating the SVM, the accuracy, sensitivity, specificity, positive predictively, and the area under curve (ROC) of the receiver operating characteristic of the trained SVM were calculated for subjects in the test sets.

### Hub Node Identification

We calculated betweenness centrality and eigenvector centrality graph measures of the rs-fMRI brain network to identify hub regions. It is noteworthy that the number of hub nodes can be highly influenced by several factors, including type(s) of the centrality measure, and the value of threshold applied on the adjacency matrix. We selected the betweenness centrality and eigenvector centrality graph measures because these measures were more frequently selected by our SFC algorithm compared to other centrality measures. In addition, these measures conceptually aligned with the integrative role ascribed to hubs, as they reflect the diversity of a region's cross-network connections. Brain regions (i.e., nodes) with betweenness centrality or eigenvector centrality larger than mean plus two standard deviation across all nodes were identified as hub nodes. Since the values of graph measures depend on the level of threshold applied on the adjacency matrix, we calculated the centrality measures by using the threshold in a range from 0.1 to 0.3 with a step of 0.01 (21 thresholds). For each value of the threshold, the betweenness centrality and eigenvector centrality of nodes were calculated and then hub nodes were identified. Next, a percentage for identification of a node as a hub node in different threshold values was calculated. Finally, we reported hub nodes that were identified in more than 85% of thresholds.

## Results

### Three- and Four-Group Classification

**Figure 2:**
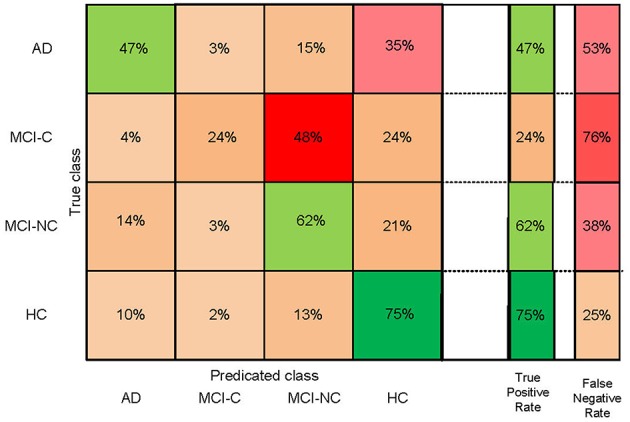
Confusion matrix of four-group (AD, HC, MCI-C, and MCI-NC) classification based on the SFC feature selection algorithm.

We performed three- and four-group classification using the SFC and DCA feature selection algorithms. Our results revealed that SFC outperforms DCA for feature selection in three- and four-group classification with an extra accuracy >7% (Table 2). The accuracies of SVM with SFC feature selection algorithm for three-group classification (“AD, MCI-C, MCI-NC” or “MCI-C, MCI-NC, HC”) and four-group classification (“AD, MCI-C, MCI-NC, HC”) were ~66 and 56%, respectively (accuracy by chance is 33 and 25%, respectively). The sensitivity, specificity, positive predictive value (PPV) and AUC of SFC algorithm are listed in Table 3, and the confusion matrix is shown in Figure 2. Our algorithm is very specific (>96%) but not sensitive (24%) in identifying MCI-C patients. In fact, the majority of miss-classified MCI-C patients (48%) were identified as MCI-NC, which indicates similarity of the brain network and structural abnormalities of MCI-C patients with that of MCI-NC patients. Our proposed method has a good sensitivity (62%) and specificity (72%) for identifying MCI-NC patients. The majority of miss-classified MCI-NC patients (21%) were identified as HC, which points to a mild abnormalities of the brain of MCI-NC patients compared to that of normal aging subjects.

**Table 2:** Accuracy of three- and four-group classification using the SFC and DCA feature selection algorithms.

Three-group classification (AD, MCI-C and MCI-NC)	SFC DCA	67.6% 57.6%
Three-group classification (HC, MCI-C and MCI-NC)	SFC DCA	66.0% 58.2%
Four-group classification (AD, HC, MCI-C and MCI-NC)	SFC DCA	56.1% 48.8%

**Table 3:** Sensitivity, specificity, positive predictive value (PPV), AUC, and accuracy of three- and four-group classification based on the SFC feature selection algorithm.

		**Three group classification (AD, MCI-C, MCI-NC)**	**Three group classification (MCI-C, MCI-NC, HC)**	**Four group classification (AD, MCI-C, MCI-NC, HC)**
Sensitivity (%)	AD	52.3	–	46.1
	MCI-C	36.0	44.0	24.0
	MCI-NC	89.6	71.7	61.8
	HC	–	69.5	75.5
Specificity (%)	AD	91.1	–	85.0
	MCI-C	97.7	90.8	96.1
	MCI-NC	47.5	74.7	72.0
	HC	–	72.6	66.3
PPV (%)	AD	77.3	–	49.7
	MCI-C	85	52.8	76.6
	MCI-NC	67.3	75.6	65.5
	HC	–	63.5	53.5
AUC	AD	0.72	–	0.65
	MCI-C	0.67	0.68	0.60
	MCI-NC	0.69	0.74	0.66
	HC	–	0.72	0.70
Accuracy (%)	AD	53	–	47
	MCI-C	36	44	24
	MCI-NC	89	72	62
	HC	–	69	75
Number of selected features	rs-fMRI	12	25	25
	sMRI	8	5	7

*The average number of selected rs-fMRI and sMRI features (across 5-folds of training data) by the SFC algorithm are listed in two bottom rows*.

### Important Features for Four-Group Classification

**Figure 3:**
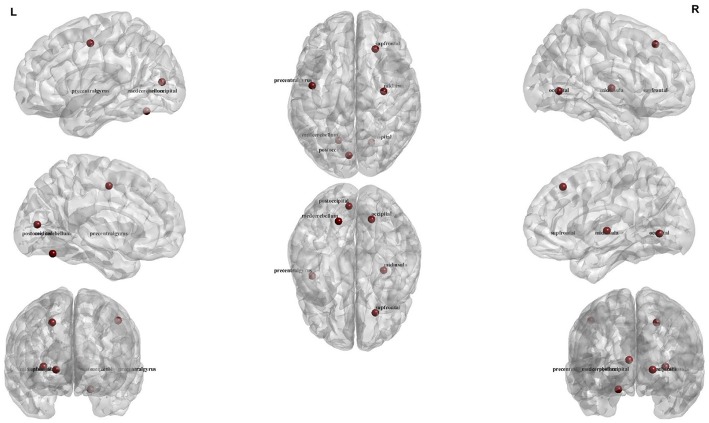
Illustration of the six regions corresponding to top six rs-fMRI features for four-group (AD, HC, MCI-C, and MCI-NC) classification.

**Figure 4:**
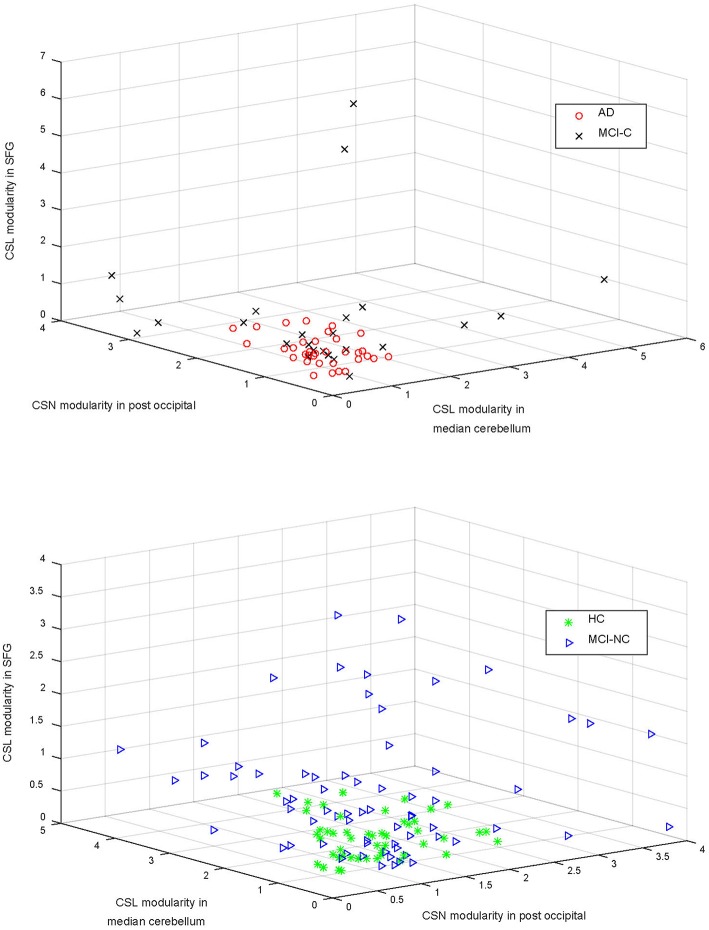
The community structure Louvain (CSL) modularity and community structure Newman (CSN) modularity in three areas (i.e., superior frontal gyrus—SFG, median cerebellum, and post occipital cortex) are compared in the top and bottom panels for AD vs. MCI-C and HC vs. MCI-NC, respectively. Modularity of the brain network in these regions were significantly different in four groups (*P* < 0.01; Table 4).

Top features that were selected by the SFC feature selection algorithm in at least 80% of training folds were listed in Table 4 that consisted of only rs-fMRI graph measures. We, however, found that eight sMRI features, including thickness of five cortical areas, were selected by the SFC algorithm in at least 60% of training folds. The top six features listed in Table 4 correspond to the rs-fMRI graph measures and represent modularity of the brain network in six brain regions (Figure 3). We used analysis of variance (ANOVA) and found a significant between-group difference (*P* < 0.01) in three features, i.e., community structure Louvain in medial cerebellum and superior frontal cortex and community structure Newman in post occipital (Table 4). Values of these three features in AD *vs*. MCI-C and HC *vs*. MC-NC are plotted in Figure 4. Results of this figure show that values of these features are clustered in AD and HC but are scattered in MCI-C and MCI-NC, indicating a similar modularity of the brain network across subjects in AD or HC group but a diverse modularity in patients with MCI corresponding to different rates of dementia in these patients.

**Table 4:** Top six features selected by the SFC algorithm for the four-group (AD, HC, MCI-C, and MCI-NC) classification.

**rs-fMRI graph measure**	**Brain area**	***P*-value**
CSL modularity	Median cerebellum	8.7 × 10^−5^
CSN modularity	Post occipital	5 × 10^−4^
CSL modularity	Superior frontal cortex	8.9 × 10^−3^
CSN modularity	Occipital	1.1 × 10^−2^
CSL modularity	Middle insula	1.4 × 10^−2^
CSN modularity	Precentral gyrus	2 × 10^−1^

*P-values were calculated using the analysis of variance (ANOVA) to find a significant difference among four groups of subjects*.

*CSL, Community structure Louvain; CSN, Community structure Newman*.

### Hub Analysis

Hub nodes of the rs-fMRI brain network in four groups of subjects (AD, MCI-C, MCI-NC, and HC) are listed in Table 5. We identified 11 nodes as the hub nodes based on two centrality measures, betweenness centrality, and eigenvector centrality, in AD (6 hubs), MCI-C (5 hubs), MCI-NC (2 hubs), and HC (2 hubs). Patients with AD and MCI-C had a common hub in the basal ganglia, and this region was not a hub in MCI-NC and HC. MCI patients (but not AD and HC) had a hub in the parietal cortex. Insular cortex was a common hub in AD, MCI-C, and HC. On the other hand, there were group-specific hubs in each group (e.g., anterior cingulate cortex in AD, occipital cortex in MCI-C, precentral gyrus in MCI-NC, and posterior cingulate in HC) which were not identified in other groups as a hub node. It is interesting to mention that 4 out of 6 features with the most discriminant information in four-group classification (Table 4) were associated with four hub regions (i.e., insular cortex, occipital cortex, cerebellum, and precentral gyrus) listed in Table 5.

**Table 5:** The rs-fMRI hub nodes in four groups of subjects (AD, HC, MCI-C, and MCI-NC).

**Hub nodes**	**AD**	**MCI-C**	**MCI-NC**	**HC**
Basal ganglia	X	X	–	–
Temporal	X	–	–	–
Anterior cingulate cortex (ACC)	X	–	–	–
Medial frontal cortex (mFC)	X	–	–	–
Thalamus	X	–	–	–
Parietal	–	X	X	–
Insula	X	X	–	X
Cerebellum	–	X	–	–
Occipital	–	X	–	–
Precentral gyrus	–	–	X	–
Posterior cingulate	–	–	–	X

*The listed areas are based on the Dosenbach atlas*.

## Discussion

We proposed a machine learning algorithm to classify patients in the early stage of AD (MCI-C and MCI-NC), patients with AD, and normal aging subjects (HC) by integrating rs-fMRI and sMRI data. This study provided three main results: (1) we examined the capability of integrating rs-fMRI and sMRI in a bi-modal approach to identify conversion from MCI to AD by evaluating performances of three- and four-group classifications (AD, MCI-C, MCI-NC, and HC); (2) we evaluated performances of SFC and DCA feature selection algorithms in identification of optimal features from a large number of rs-fMRI and sMRI features. Our results revealed that the SFC algorithm outperformed the DCA feature selection algorithm by providing an extra accuracy of >7% in four-group classification; and (3) we identified hub nodes of the rs-fMRI brain network in AD, MCI-C, MCI-NC, and HC, and found different hubs in patients within the early stage of AD.

Prediction of the early stage of AD using rs-fMRI and sMRI data based on a four-group classification (AD, MCI-C, MCI-NC, and HC) remains a relatively unexplored task. We performed three- and four-group classifications by integrating rs-fMRI and sMRI features, and observed that combining structural and functional MRI features improves performance of classification. We demonstrated in our previous study that a bi-modal (sMRI and rs-fMRI) approach outperformed a unimodal (sMRI or rs-fMRI) approach for a two-group classification (MCI-C and MCI-NC) with an increased accuracy up to 17% (11). Therefore, we decided to use a bi-modal approach (sMRI and rs-fMRI) for three- and four-groups classification in the current study. Schouten et al. utilized functional and structural MRI and classified 16 AD patients from 22 healthy controls, found that combining features of two modalities improves performance of classification, and achieved an accuracy of 89.5% in two-group (AD *vs*. HC) calcification (54). In another study, Canu et al. combined features extracted from structural MRI (cortical thickness in 68 cortical regions) and diffusion tractography (white matter microstructure) to classify 62 early onset AD and 27 behavioral variant of frontotemporal dementia patients, and reported 82% classification accuracy by integrating features of two modalities (55). Suk et al. integrated sMRI and positron emission tomography (PET) features in a deep learning algorithm, and reported 98.8, 90.7, 83.7, and 83.3% accuracies in binary (two-group) classification AD/HC, MCI/HC, AD/MCI, and MCI-C/MCI-NC, respectively (56). It is noteworthy that Suk et al. did not test performance of their proposed algorithm in three- or four-group classification, as we did. To our knowledge, this is the first study which integrated rs-fMRI with sMRI data in a four group (AD, MCI-C, MCI-NC, and HC) classification approach. For the binary classification MCI-C *vs*. MCI-NC, Zhang et al. (57) achieved 73.9% accuracy by utilizing a multi-modal neuroimaging approach using FDG-PET, sMRI, and cerebrospinal fluid (CSF) data. Beheshti et al. used sMRI features in a discriminative feature ranking method to find the most discriminative feature set, and reported 75% accuracy in MCI-C vs. MCI-NC binary calcification (9). In another study, Young et al. provided AD prediction model by adding the apolipoprotein E (ApoE) genotype to FDG-PET, sMRI, and CSF data, and reported 74% accuracy in classification of MCI-C vs. MCI-NC (58). It is noteworthy that previous studies have shown that MCI is a heterogeneous condition where MCI-NC subjects appear more healthy and MCI-C subjects appear more in AD condition (59).

Figure 2 shows that our algorithm was more accurate in identification of HC compared to other groups of subjects, which is expected due to the similarity of the brain structure and network of patients with MCI and AD compared to that of HC. The best and worst accuracies of our algorithm were 75% for HC and 24% for MCI-C groups, respectively (Figure 2). Results in Table 3 show that our algorithm was specific (>90%) but not sensitive (<44%) in identifying MCI-C group compared to other groups, in both three- and four-group classification. The majority of miss-classified MCI-C patients were identified as MCI-NC patients (Figure 2), indicating similarity of the rs-fMRI and sMRI features of patients in the former group with that of the latter group. On the other hand, our algorithm had a good sensitivity and specificity in identifying MCI-NC patients.

Identifying MCI-C patients is a difficult task since we used the baseline rs-fMRI and sMRI data in these patients and they converted to AD 6 to 36 months after that. In addition to using the baseline data in these patients, they had a heterogeneity in their conversion time to AD from 6 to 36 months. MCI-C patients who converted to AD in a longer time (e.g., at 36 months) after baseline may have a similar brain network and structure at baseline compared to MCI-NC patients, who did not convert to AD. On the other hand, brain network and structure of the MCI-C patients who converted to AD in a shorter time (e.g., at 6 months) may be similar to that of the AD patients. Furthermore, MCI-C patients were the only unstable group of patients who had a change of status from MCI to AD during 36 months follow-up. In fact, subjects in HC, MCI-NC, and AD groups were stable and did not convert to another group during at least 36 months. Moreover, we observed instability in the MCI-C patients that some of them had conversion to AD and then revision to MCI during 36 months follow-up. It is noteworthy that we excluded MCI-C patients with multiple conversion and revision in our analysis. Therefore, it is expected that a classifier has a lower performance in identifying MCI-C compared to other groups. In line with this expectation and as shown in Figure 2 and Table 3, our proposed algorithm provided a superior performance in classifying HC, MCI-NC, and AD compared to MCI-C. It is noteworthy that we evaluated our algorithm in identification of the early stage of AD based on three- and four-group classification (AD, MCI-C, MCI-NC, and HC), while most previous studied investigated a two-group classification (e.g., AD vs. HC and AD vs. MCI) (5, 60, 61).

Top six features and brain regions that were selected by SFC in four-group classification are listed in Table 4 and Figure 3. These features were all related to rs-fMRI graph measures and represent modularity of the brain network in six cortical regions (median cerebellum, post occipital, superior frontal gyrus, occipital, middle insula, and precentral gyrus). The modularity was calculated based on the community structure Louvain or community structure Newman graph measures. A modular network has an arrangement of nodes in large modules such that maximum possible number of edges lies within groups and minimum possible number of edges lies between groups (62). Modularity of the brain network has been showed to be informative in the early stages of the neurodegenerative disease (63). A recent study demonstrated the ability of modularity of the brain network in discriminating AD and HC (63). Results of ANOVA in Table 4 show that the modularity in three brain regions, i.e., median cerebellum, post occipital and superior frontal gyrus, were significantly different in four groups of subjects (*P* < 0.01). In agreement with our results, previous studies reported association of posterior occipital, superior frontal cortex, occipital, and middle insula with AD (64–66). Another observation from Table 4 is that there was no sMRI feature among the top six features which may indicate that rs-fMRI is more informative than sMRI in identification of the early stage of AD. This observation was in agreement with previous studies reported that biomarkers based on the functional brain network may outperform biomarkers based on the structural measures in predicting the early stage of AD (11, 67).

In Figure 4, we compared four groups based on modularity of the brain network in three regions (i.e., median cerebellum, post occipital, and superior frontal gyrus) which were significantly different in four groups (*P* < 0.01). Figure 4 shows that modularity in AD or HC was clustered across subjects but the modularity in MCI-C or MCI-NC was scattered across subjects. This scatter of modularity in patients with MCI may be related to the inhomogeneity in MCI group, as a transitional state between normal aging and AD, in that characteristic of their brain may vary between two extremes from HC to AD.

Results of our hub analyses in Table 5 are generally consistent with previous findings in terms of localization, and provide additional support for the underlying topological organization of the early stage of AD. Interestingly, some hub areas in Table 5 (i.e., insular cortex, occipital, cerebellum, and precentral gyrus) were also identified by the SFC algorithm as important areas in classification of four groups. We found that the insular cortex was a common hub node in AD, MCI-C, and HC, and our finding is in agreement with previous studies showing importance of this area in AD (68, 69). Our finding that Basal ganglia was a common hub in AD and MCI-C is in agreement with a recent study showing pivotal role of this area in patients with early-onset AD (70). We found the parietal cortex as a common hub in MCI-C and MCI-NC patients. In agreement with our finding, association of the parietal cortex with AD has been reported in previous studies (71, 72). Our results revealed that AD and MCI-C subjects had larger number of hub nodes compared to MCI-NC and HC subjects which may relate to the abnormalities in largescale network connectivity in AD and MCI-C brain regions (73).

## Data Availability

Publicly available datasets were analyzed in this study. This data can be found here: http://adni.loni.usc.edu/.

## Ethics Statement

Data used in this paper were obtained from the Alzheimer's Disease Neuroimaging Initiative (ADNI) database (http://ADNI.loni. usc.edu). Data collection and sharing for this project was funded by the Alzheimer's Disease Neuroimaging Initiative (ADNI) (National Institutes of Health Grant U01 AG024904).

## Author Contributions

SH: preparing required data from ADNI, data analysis, statistical analysis, and drafting and revision of the manuscript. AE: interpreting results and revision of the manuscript. AB-F: study design and conceptualization, data analysis and interpretation of the results, drafting, and revision of the manuscript.

### Conflict of Interest Statement

The authors declare that the research was conducted in the absence of any commercial or financial relationships that could be construed as a potential conflict of interest.

## Footnotes

^1^Data used in the preparation of this article were obtained from the Alzheimer's Disease Neuroimaging Initiative (ADNI) database (adni.loni.usc.edu) that was launched in 2003 and led by Principal Investigator Michael W. Weiner, MD. The primary goal of ADNI has been to test whether serial MRI, positron emission tomography (PET), other biological markers, and clinical and neuropsychological assessment can be combined to measure the progression of mild cognitive MCI and early AD. For up-to-date information, see www.adni-info.org.
